# Development of a novel clinical support tool for active surveillance of low risk papillary thyroid cancer

**DOI:** 10.3389/fendo.2023.1160249

**Published:** 2023-09-11

**Authors:** Eleanor White, Bridget Abbott, Geoffrey Schembri, Anthony Glover, Roderick Clifton-Bligh, Matti L. Gild

**Affiliations:** ^1^Department of Endocrinology and Diabetes, Royal North Shore Hospital, Sydney, NSW, Australia; ^2^Department of Radiology, Royal North Shore Hospital, Sydney, NSW, Australia; ^3^Faculty of Medicine and Health, University of Sydney, Sydney, NSW, Australia; ^4^Department of Endocrine Surgery, Royal North Shore Hospital, Sydney, NSW, Australia

**Keywords:** active surveillance, low risk thyroid cancer, clinical support tool, papillary thyroid cancer, watchful waiting, de-escalation treatment

## Abstract

**Background:**

Active surveillance (AS) is an alternative to surgery in select patients with very low risk papillary thyroid cancer (PTC). Many clinicians feel ill-equipped in selecting appropriate patients. We aimed to 1) Develop an evidence-based web delivered decision support tool to assist clinicians in identifying patients appropriate for AS; and 2) Evaluate the prevalence of patients suitable for AS in a tertiary high volume thyroid cancer centre.

**Method:**

A REDCap web based clinical support tool was developed utilising evidence-based characteristics for AS suitability available to clinicals during initial assessment. A retrospective database was interrogated for patients who underwent hemithyroidectomy between 2012 – 2021 with final histopathology demonstrating PTC. Patients with PTCs>2cm, missing data, benign disease on surgical histopathology or incidental PTC were excluded.

**Results:**

Between 2012 - 2021, 763 patients underwent hemithyroidectomy with final histopathology confirming PTC. Of these, 316 patients were excluded (missing data, incidental PTC, concomitant hyperparathyroidism were most common reasons for exclusion) and 114/447 remaining patients had a pre-operative fine needle aspirate (FNA) of Bethesda V or VI (high likelihood of malignancy). Using the tool, 59/114 (52%) met criteria for AS. The majority of patients were female (85% *vs* 15% male); median age 36 years (range 19 – 78). Following initial surgery, 10/59 patients had a completion thyroidectomy, with 4/10 demonstrating malignancy in contralateral lobe and eight of those patients undergoing I^131^ ablation. During a median follow up of over 3 years, 49/59 (83%) did not require further surgery or intervention with no patients developing recurrence. A subgroup analysis with second radiology assessment excluded 4/59 patients as meeting criteria for AS based on presence of ETE on preoperative ultrasound. None of these 4 patients had completion thyroidectomy.

**Conclusion:**

Our clinical support tool identifies patients with PTC potentially suitable for AS which could be utilised during initial patient assessment. In a retrospective cohort of patients who had hemithyroidectomy for PTC with a pre-operative FNA diagnosis of Bethesda V or VI, 55/114 (48%) patients may have been suitable for AS. Prospective validation studies are required for implementation of the tool in clinical practice.

## Introduction

Incidence of thyroid cancer has increased over past decades, largely attributed to improvement in imaging techniques and identification of incidental thyroid nodules (1). Most of these nodules will be benign; however, a small percentage will harbour malignancy (1). Papillary thyroid cancer (PTC) is the most common type of thyroid malignancy, with excellent prognosis and 5 year survival rates over 97% (2, 3). Despite increased diagnosis of incidental thyroid cancers, mortality rates remain largely unchanged and are overall low, suggesting over-diagnosis of low risk cancers (4). In addition to the psychological impact of a cancer diagnosis, there are financial implications on the health care industry for overdiagnosis which need to be considered. In South Korea the economic burden of thyroid cancer increased from $257 million in 2000 to $1.7 billion by 2010, with no change in thyroid cancer mortality (5). These figures are similarly seen in other countries and highlight the need to balance the risk and cost of treatment against the risk of disease progression (6).

Thyroid cancer can be classified into low, intermediate or high risk based on tumour stage, presence of lymphovascular invasion (LVI), extrathyroidal extension (ETE) and tumour location in relation to the capsule (7). Low risk papillary thyroid cancer are smaller tumours, typically less than 1cm (microcarcinoma), without LVI, ETE or presence of nodal or distant metastasis (8). Certain histological subtypes of papillary thyroid cancer or presence of molecular mutations such as *BRAF V600E* can behave more aggressively, and this information is typically identified in the final histopathology report (2).

The natural history of low risk PTC is indolent and slow growing, with low rates of locoregional or distant metastasis (9). Historically the management of these cancers has been surgical, with either total thyroidectomy or lobectomy. Following the updated American Thyroid Association (ATA) guidelines in 2015, hemithyroidectomy is now an accepted surgical option for low risk differentiated cancer (7). Potential surgical complications of hemithyroidectomy such as wound infection, vocal cord or recurrent laryngeal nerve palsy, temporary or permanent hypoparathyroidism, or hypothyroidism may still occur and while these rates are low in experienced centres, these should be considered in treatment decisions (10, 11).

Due to this epidemic of overdiagnosis, the role of active surveillance (AS) as an alternative to thyroid surgery in the management of low risk PTC has gained traction in the past decade and is included in the most recent ATA guidelines (7). Evidence for active surveillance dates back to 1993, through a prospective study in Kuma Hospital where patients with low risk PTC either underwent active surveillance or immediate surgery (12). Patients in the AS arm had low rates of tumour growth or development of nodal disease over 10 years. Those who had tumour growth or nodal disease underwent rescue surgery with no difference in mortality between groups (12). This safety data has now been replicated by many other centres (13–15). The bulk of studies for AS are in papillary microcarcinomas (PMC) however there is now evidence for inclusion of tumours up to 2cm. Sakai et al. included patients with tumours up to 2cm in their prospective active surveillance trial, demonstrating low rates of tumour growth and lymph node metastases in patients who underwent active surveillance, at 7% and 3% respectively (13). Even patients who underwent subsequent surgery from the AS arm did not demonstrate any disease recurrence in the follow up period, reflecting that the size criteria can be safely extended (13). This data has also been replicated by Ho et al. (2022) (14).

While there is robust evidence for AS for low risk PTC <2cm, not every patient with a low risk PTC should undergo AS. Characterising both the tumour and patient features that make patients ideal candidates has become clearer in the recent years. Tuttle et al. have described tumour and patient characteristics that help risk stratify patients into ideal, appropriate, or inappropriate for active surveillance (15). Presence of nodal or distant metastases, extrathyroidal extension (ETE) or lymphovascular invasion (LVI), or unfavourable tumour location (e.g. adjacent to a critical structure with potential for tumour invasion) exclude active surveillance as a suitable option (15). Lohia et al. recommended there should be >2mm of normal parenchyma between the thyroid nodule and the capsule to avoid potential tumour growth to nearby critical structures and this has been adopted by some centres (15).

Despite the continuously growing body of evidence for active surveillance, many clinicians do not feel comfortable in identifying appropriate patients (16). Wei et al. performed a scoping review to explore factors that influenced clinician and patients decision in selecting either active surveillance, hemi- or total thyroidectomy for low risk thyroid cancer (17). Patients were more likely to consider conservative therapy if provided with adequate information (17). Key barriers identified that prevented patients or physicians from selecting AS included lack of published surveillance protocol/guidelines and physician comfortability in recommending AS to patients (17, 18). This highlights the potential role for a clinical decision support tool to help clinicians identify if their patient is suitable for active surveillance, which to our knowledge is not available in the literature.

## Methods

This study was performed at the Department of Diabetes and Endocrinology, the Department of Endocrine Surgery and the Department of Radiology at Royal North Shore Hospital. Ethics approval was by the local Northern Sydney Health District Ethics Committee (2020/ETH02787).

### Clinical support tool development

A web based clinical support tool was developed and built on REDCap software (**Supplementary Appendix 1**). It utilised evidence based characteristics for AS suitability and generated a result indicating whether a patient was suitable for active surveillance. The tool was designed to apply the pre-operative ultrasound findings and patient data such as presence of local or distant metastasis, age, calcitonin level, comorbidities that would preclude them from surgery (15).

**Figure 1:**
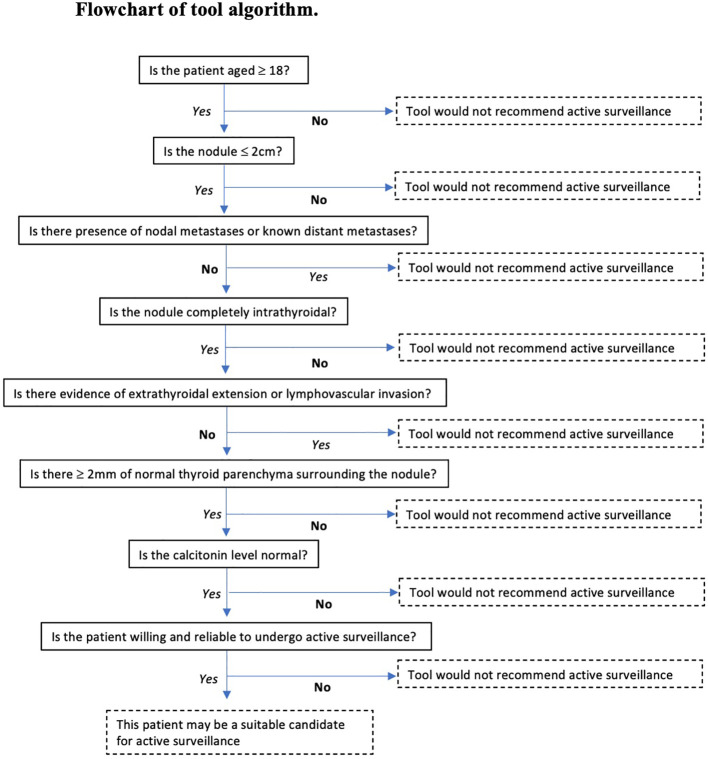
The flowchart outlines the algorithm of the clinical support tool.

Patients were deemed suitable for active surveillance by the tool if they were aged ≥18 years, tumour size ≤2cm, no evidence of ETE or LVI on ultrasound, had no nodal or distant metastases and >2mm normal gland parenchyma (**Figure 1**). Tumour size was determined by the maximum tumour diameter and tumours up to 2cm were included.

### Retrospective analysis

A retrospective thyroid cancer surgery database was interrogated for patients who underwent hemithyroidectomy between 2012-2021 with final histopathology demonstrating PTC. This database captures information about thyroid cancer surgery performed by endocrine surgeons at our institution and their affiliated operating sites.

Patients were excluded from analysis if; there was missing histopathology or pre-operative ultrasound data, the histopathology demonstrated a cancer other than PTC, the final histopathology showed an incidental PTC, they had hemithyroidectomy and contralateral nodulectomy, or if they had concomitant hyperparathyroidism and underwent parathyroidectomy.

Remaining patients were stratified according to pre-operative Bethesda category. Our cohort of interest were patients with a high risk of malignancy on biopsy or with a pre-operative biopsy result of either Bethesda V (BV, suspicious for malignancy) or Bethesda VI (BVI, malignancy). The tool was applied utilising pre-operative ultrasound reports and information from surgical database forms. Information was also extracted from surgical consultation letters, histopathology and imaging results, referral letters and electronic medical records where available. Given the retrospective nature of this study, we were unable to ascertain patient willingness to participate in active surveillance, however for the purpose of our prevalence assessment we assumed all patients would be amenable.

### Subgroup analysis with sonologist

A sonologist and imaging trainee reviewed the ultrasound images in a subgroup of 22 patients to determine if AS features were easily identified on pre-operative ultrasound. The preoperative ultrasound images were unavailable for the remaining 37 patients deemed suitable for AS. The nodule of interest was assessed for presence of LVI, ETE, proximity to critical structures and presence of 2mm normal gland surrounding the nodule, and the tool was applied.

### Statistical analysis

Statistics were performed in Microsoft Excel. Sample T tests were used to analyse differences in age, and median tumour size between the group had completion surgery versus those who had no completion surgery as well as between the subgroup who had evidence of malignancy on completion surgery versus those who had no malignancy. A p value of <0.05 was considered significant.

## Results

### Prevalence of AS suitability

**Figure 2:**
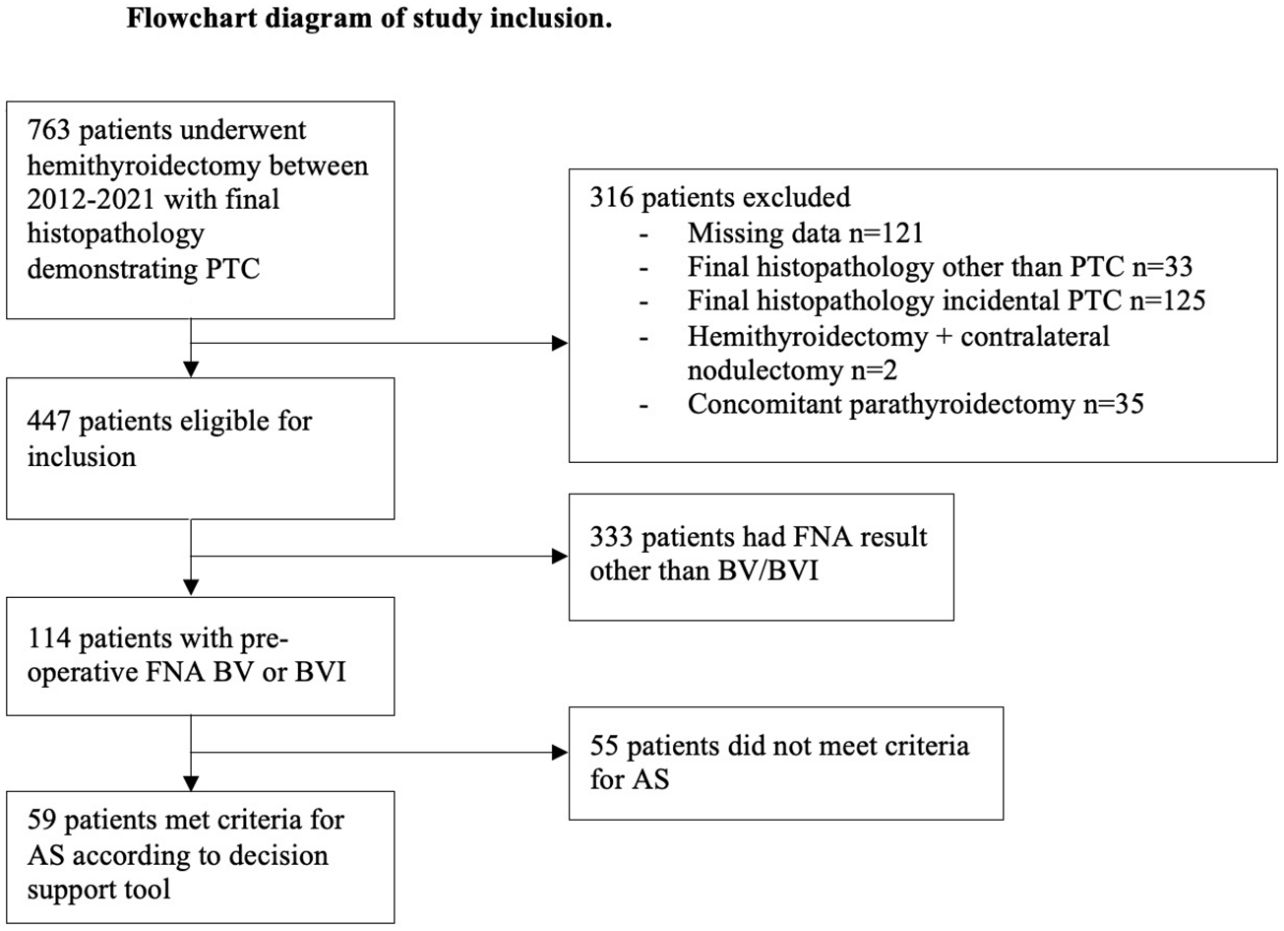
The PRISMA diagram outlines the selection process for our study. PTC, papillary thyroid cancer; FNA, fine needle aspirate; AS, active surveillance; BV, Bethesda V; BVI, Bethesda VI.

During 2012 – 2021, 763 patients underwent hemithyroidectomy with final histopathology demonstrating PTC. Excluded from analysis were 316 patients (see **Figure 2**), leaving a cohort of 447 potentially eligible patients. We analysed the cohort of 447 patients by Bethesda category and found 114 patients (26%) had a pre-operative Bethesda result of BV or BVI. The tool was applied to the potentially suitable 114 patients with BV/BVI FNA and 59/114 (52%) were found to meet criteria for active surveillance. Of the 55/114 (48%) who were deemed unsuitable, all patients had nodule size >2cm. Assessment of all Bethesda results yielded: 353/447; 79% were unsuitable (>2cm tumour) and 95/447 (21%) potentially suitable for AS. 63% of those 95 patients were BV/VI reflecting that a majority would have a high risk of malignancy from the FNA.

### Characteristics of patients suitable for AS

Most patients with BV/BVI results who were deemed suitable for active surveillance were young females, with median age 36 years (**Table 1**). Median size of nodules in this group was 10mm, with size ranging 4mm – 19mm and majority of patients had T1a tumours (tumour ≤1cm).

**Table 1:** Baseline characteristics of patients suitable for active surveillance.

Characteristic	Value
Age range	19 – 78 years
Median age	36 years
Male	9/59 (15%)
Female	50/59 (85%)
Bethesda V	29 (49%)
Bethesda VI	30 (51%)
Nodule size range	4-19mm
Nodule size	T1aN0M0 – 37/59 (63%) T1bN0M0 – 22/59 (37%)
Median nodule size	10mm

TNM refers to American Joint Committee on Cancer (AJCC) staging tumour (T), nodes (N), metastes (M).

### Patients who had further surgical intervention

We next analysed the characteristics of the cohort who went on to have further surgical intervention to ascertain if we could predict the need for further treatment. Most patients (49/59) did not require any further surgical or medical intervention for a median of 3.25 years (ranging from 0.2 – 7.35 years) follow up. After MDT and patient discussion, ten patients went on to have completion hemithyroidectomy. Evaluation of their initial histopathology demonstrated higher rates of lymphovascular invasion (80% versus 18%, p<0.01) and lymph node involvement (90% versus 27%, p<0.01) compared to patients who did not undergo completion hemithyroidectomy. There was a trend for patients who underwent completion surgery to be younger (median age 34 *vs*. 39 years, p=0.05) with bigger nodules (median nodule size 12mm *vs*. 10mm, p=0.05) however these were of borderline statistical significance. Rates of tumour multifocality or presence of ETE did not differ significantly between groups (**Table 2**).

**Table 2:** Comparison of patients who had no completion surgery vs. completion surgery.

Characteristic	No completion surgery (n=49)	Completion surgery (n=10)	P value
Age range	19 – 78	20 – 52	
Median age	39 years	34 years	0.05
Male Female	8/49 (16%) 41/49 (84%)	1/10 (10%) 9/10 (90%)	0.619
Pre-operative FNA Bethesda V Bethesda VI	23/49 (47%) 26/49 (53%)	6/10 (60%) 4/10 (40%)	
Median nodule size (range)	10mm (4-19)	12mm (8-18)	0.058
Tumour focality Unifocal Multifocal	41/49 (84%) 8/49 (16%)	7/10 (70%) 3/10 (30%)	0.319
Presence of ETE	9/49 (18%)	2/10 (20%)	0.905
Presence of LVI	9/49 (18%)	8/10 (80%)	<0.01
Presence of LN involvement	13/49 (27%)	9/10 (90%)	<0.01
BRAF V600E positivity	27/49 (55%)	8/10 (80%)	0.149

FNA, fine needle aspirate; ETE, extrathyroidal extension; LVI, lymphovascular invasion; LN, lymph node.

### Patients with malignancy on completion thyroidectomy

We next examined the characteristics of patients who were found to have malignancy in the contralateral lobe and this was seen in 4/10 (40%) of patients who underwent completion thyroidectomy. In these patients the average age was 36 years and median tumour size was 11mm. All four followed up with I-^131^ therapy, and four patients with benign histopathology in contralateral lobe also received I-^131^ (**Table S1**). No patients in the study cohort had evidence of disease recurrence at median 3.25 years follow up (0.2 – 7.35 years). Evaluation of the initial hemithyroidectomy histopathology of the four patients with contralateral lobe malignancy demonstrated that all had classical papillary architectures subtype. All four had lymph node involvement on initial histopathology (**Table 3**). Two of the four patients with malignancy in contralateral lobe had multifocal tumours in initial histopathology, compared with one out of the six patients with benign histopathology on completion. No patients with malignancy in contralateral lobe had an aggressive histological subtype (such as poorly differentiated features, tall cell variant, diffuse sclerosing etc).

**Table 3:** Characteristics of initial nodule size, histopathology, further treatment and recurrence in patients who had completion thyroidectomy.

Pt	Age	Sex	Nodule size	Initial histopathology	Completion Histopathology	I ^131^ therapy	Recurrence
1	20	F	12mm	Unifocal 12mm tumour, classical, ETE, LVI, 11/17 LN involved, BRAF V600E positive	Benign	No	No
2	35	F	13mm	Unifocal 14mm tumour, classical, no LVI, ETE, 3/4 LN involved, tracheal invasion, BRAF V600E positive	0.4mm deposit in contralateral lobe, no LN	Yes – 224MBq	No
3	52	F	18mm	Unifocal 19mm tumour, classical, no LVI, no ETE, 0/6 LN involved, BRAF not done	Benign	Yes – 1.8 GBq	No
4	30	F	12mm	Unifocal 12mm tumour, classical, LVI, no ETE, 2/4 LN involved, BRAF V600E positive	Benign	Yes – 4 GBq	No
5	33	F	8mm	Unifocal 12mm tumour, classical, LVI, no ETE, 1/7 LN involved, BRAF V600E positive	Benign	Yes – 1 GBq	No
6	36	M	12mm	Multifocal 13mm tumour, classical, LVI, no ETE, 2/3 LN involved, BRAFV600E positive	2mm papillary thyroid cancer, no LN involved	Yes – 1 GBq	No
7	37	F	14mm	Unifocal 15mm tumour, mixed papillary/follicular, LVI, no ETE, 1/2 LN involved, BRAF V600E positive	Benign	Yes – 1 GBq	No
8	32	F	14mm	Multifocal 11mm tumour, mixed papillary/follicular, LVI, no ETE, 5/14 LN involved, BRAF V600E positive	Benign	No	No
9	32	F	11mm	Unifocal 8mm tumour, classical, no LVI, no ETE, 3/5 LN involved, BRAF V600E negative	Multifocal PTC, largest deposit 9mm, no LVI, no ETE, 3/5 LN involved	Yes – 4 GBq	No
10	41	F	9mm	Multifocal 9mm tumour, classical, LVI, no ETE, 1/4 LN involved, BRAF V600E positive	6mm PTC, no LN	Yes – 4 GBq	No

ETE, extrathyroidal extension; LVI, lymphovascular invasion; LN, lymph node; PTC, papillary thyroid cancer; MBq, megabecquerel; GBq, gigabecquerel.

Histopathology from initial hemithyroidectomy showed similar lesion size between patients who had malignancy *vs*. benign pathology on completion hemithyroidectomy, with median size 11mm *vs*. 12mm respectively. Patients who went on to have malignancy in contralateral lobe were slightly younger, with median age 32.5 years versus 35.5 years. There were similar rates of ETE in each group however higher rates of LVI in the group who did not have evidence of malignancy in contralateral lobe (**Table 3**).

### Imaging subgroup

To investigate the real world application of this tool, we conducted a subgroup analysis of patients in whom we were able to obtain their pre-operative ultrasound. Of the 59 patients who met criteria for active surveillance, the preoperative ultrasound images were available for 22 patients. None of the patients in this subgroup analysis were patients who had completion thyroidectomy. In 21/22 patients (95%), the sonologist was unable to identify >2mm of normal gland parenchyma between the nodule and the capsule in the saved images, identifying a real world limitation of this tool. Nodule size ranged from 5-14mm, with median nodule size of 9mm. ETE on pre-operative ultrasound was defined as extension of the nodule beyond the normal capsule line. The sonologists identified 4/22 patients with evidence of ETE which had not been identified on initial ultrasound report. Of these 4 patients, 1 was identified as definite ETE and 3 were deemed to have features suggestive of ETE, rendering them unsuitable for AS according to our protocol.

## Discussion

In this retrospective analysis of patients treated by hemithyroidectomy for nodules with pre-operative cytology BV or BVI FNA, we have shown a substantial proportion of patients may have been suitable for active surveillance rather than surgery. The strongest evidence for active surveillance is for patients with high risk of malignancy (BV or BVI cytology) and as there is limited data for active surveillance in other Bethesda categories, we focused our analysis on patients with pre-operative FNA of BV or BVI. To our knowledge, there are no decision support tools available to help clinicians identify patients suitable for AS. We present a novel tool with real world application, outlining the experiences of using this tool in a cohort from a tertiary centre.

The demographic results for our appropriate AS cohort were unsurprising. Most patients in our suitable active surveillance cohort were young females which reflects known epidemiological data (19). A strength of this analysis is that it includes patients with tumours up to 2 cm. 37% of patients who were suitable for active surveillance had tumours >1 cm, and therefore would have been excluded without the extended 2 cm tumour diameter. There was no control group for comparison as very few patients in our cohort have AS thus far and the database only captures patients post surgery.

It is reasonable to propose that patients who required further surgery or medical intervention had progressive disease either distant metastases or further lymph node disease. While we do not know if these patients would have progressed without the initial surgery, our tool correctly identified characteristics meeting criteria for AS with the majority (49/59) having no further surgery. Patients who had completion surgery were generally younger, with lymph node involvement and presence of lymphovascular invasion on initial histopathology. In the group who did not have completion, we are unable to ascertain whether there was malignancy in the remaining lobe however it is reassuring that there has been no evidence of disease recurrence or development of nodal or distant metastases in this cohort (as evidenced by lack of further interventions and available progress imaging reports). Of the patients who had completion thyroidectomy, the majority (7/10 patients) had tumour size >1cm. No patients who had evidence of malignancy on completion thyroidectomy had evidence of disease recurrence on available follow up data, however we are unable to ascertain if any these patients went on to have further treatment at another treatment location. Tumour size, multifocality, presence of ETE or LVI, presence lymph node involvement and BRAFV600E positivity did not predict malignancy in contralateral lobe. As hemithyroidectomy as a first line operation for low risk cancer gains further evidence, there is a trend away from completion thyroidectomy unless initial histopathology has concerning features. This study was not able to identify any factors in this cohort that predicted risk of malignancy in contralateral lobe, though a key limitation is the small sample size. The higher rates of LVI in the group who had completion thyroidectomy who did not have malignancy in contralateral lobe is of doubtful relevance and likely due to small sample size.

We next attempted to understand how the tool could work in clinical practice, and the feasibility of interpreting findings from the ultrasound report and original images and incorporating these into this tool. Our subgroup analysis with our imaging specialists highlighted some of the challenges of the real world application of this tool. Firstly, there is inter-observer variability in detecting the presence of ETE on ultrasound. Of the 4 patients in whom our imaging specialists deemed there to be ETE present, there was no comment on ETE in the original ultrasound report in three patients and in one patient it was documented as not present. Final histopathology for these patients demonstrated two of four had no ETE and two had presence of ETE, reflecting ultrasound and pathological disparity. The sonologist reviewing the subgroup analysis had 40 years of experience. We conclude that ETE is not feasible to be routinely analysed in a pre-operative ultrasound due to significant heterogeneity, and in further refinement of this tool we would focus more on tumour location in relation to critical structures. Several features we have used to assess feasibility for active surveillance on preoperative ultrasound (ETE, LVI, presence of 2 mm normal gland around nodule) are not routinely reported and a training and reporting template when looking at these ultrasounds may be beneficial.

Furthermore in our subgroup analysis, 21/22 patients did not have ≥2 mm between the nodule and capsule on the ultrasound. However, nodule position is inconsistently reported in ultrasound results, making it difficult to interpret from the report alone whether a nodule would meet this criteria and thus this represents a limitation to the application of this tool. Collaboration with our imaging colleagues would be necessary to develop more relevant reporting schema. Absence of 2mm of normal gland parenchyma does not preclude suitability for active surveillance, and future refinement of this tool would likely eliminate this specification and focus on more discriminatory markers such as tumour location in relation to critical structures and tumour volume kinetics (20).

This study has several key strengths. Firstly, it demonstrates a novel idea of creating a clinical decision support tool designed to empower clinicians in identifying patients who are potential candidates for active surveillance as a lack of physician knowledge has been identified as a barrier to AS implementation (19). Secondly, it demonstrates likely numbers of patients who would be potentially suitable for active surveillance based on the experience of a tertiary centre. In our cohort, 59/114 (52%) of patients with Bethesda V or VI were suitable for active surveillance based on our clinical support tool, with 55/114 (48%) deemed suitable following imaging review. The main factor that excluded patients from suitability for active surveillance was tumour size >2 cm.

Limitations of this study are that it is retrospective single centre data. There is selection bias given we analysed a low risk population by selecting patients who had been managed with hemithyroidectomy. Our follow up data was limited to information available through our thyroid cancer database and electronic medical record. We were unable to ascertain whether patients have gone on to receive further treatment through other centres, which may underestimate the risk of recurrence in our population. Thirdly, our imaging review of the 59 patients identified as being suitable for AS was incomplete as we only had access to the images of 22 patients. It is likely that further patients would have been excluded had we been able to review the entire cohort. Acknowledging this limitation, we have attempted to estimate prevalence of patients suitable for AS based on our available data.

Lastly, in the application of our clinical support tool, patient willingness to undertake active surveillance and engage with follow up is a key component of suitability. In applying our tool to this retrospective data, we were unable to ascertain patient willingness to undergo AS. Prospective studies are required to further evaluate the effectiveness of our clinical support tool in patients who are suitable for AS.

## Conclusion

Our clinical decision support tool is a novel method of assisting clinicians in selecting patients that are suitable for active surveillance. Utilising this tool in our single centre cohort, almost half (48%) of patients with BV/BVI FNA and PTC on final histopathology would have been suitable for active surveillance. No patients had evidence of disease recurrence throughout our follow up period. Further prospective studies are required to evaluate this tool.

## Data availability statement

The raw data supporting the conclusions of this article will be made available by the authors, without undue reservation.

## Ethics statement

The studies involving humans were approved by Northern Sydney Health District Ethics Committee. The studies were conducted in accordance with the local legislation and institutional requirements. Written informed consent for participation was not required from the participants or the participants’ legal guardians/next of kin in accordance with the national legislation and institutional requirements.

## Author contributions

EW: Methodology, data collection, formal analysis, writing -original draft, writing - reviewing and editing. BA: data analysis, writing -reviewing and editing. GS: data analysis, writing - reviewing and editing. AG: methodology, writing - reviewing and editing. MG: Supervision, conceptualisation, formal analysis, methodology, writing - reviewing and editing. RC-B: supervision, conceptualisation, methodology, writing - reviewing and editing. All authors contributed to the article and approved the submitted version.
