# Supplementary material for: Development of a novel clinical support tool for active surveillance of low risk papillary thyroid cancer
Source: Front Endocrinol (Lausanne). 2023 Sep 11;14:1160249. doi: 10.3389/fendo.2023.1160249 (PMC10520546; doi:10.3389/fendo.2023.1160249)
Supplement: Supplementary file 1 [file DataSheet_1.docx]

**Supplementary Appendix 1. Active Surveillance Tool Algorithm**

| Factor | Ideal | Appropriate | Inappropriate |
| --- | --- | --- | --- |
| Age | >60 | 18-59 | <18 |
| Solitary nodule | Yes | Yes or No | No |
| Size | <1cm | <2cm | >2cm |
| Nodule >2mm normal gland | Yes | Yes | No |
| Intrathyroidal | Yes | Yes | No |
| Invasion | No | No | Yes |
| ETE | No | No | Yes |
| Calcitonin normal | Yes | Yes | No |
| Nodal metastases | No | No | Yes |
| Distant metastases | No | No | Yes |
| Comorbidities that preclude surgery | Yes | Yes | Yes |
| Pt willingness | Yes | Yes | No |
| Reliable for follow up | Yes | Yes | No |

Permutations that lead to ideal: (ALL OF THE FOLLOWING)

- Age >60
- Solitary nodule = yes
- Nodule surrounded by >2mm normal gland = yes
- Intrathyroidal = yes
- Invasion = no

Permutations that lead to appropriate: (ALL OF THE FOLLOWING)

- Age >18
- Solitary nodule = yes or no
- Nodule surrounded by <2mm normal gland = yes
- Intrathyroidal = yes
- Invasion = no

Permutations that lead to inappropriate: (ANY OF THE FOLLOWING)

- Age <18
- Intrathyroidal = no
- Invasion = yes
- ETE present = yes
- Nodal metastases present = yes
- Distant metastases present = yes
- Reliable - “No”
- Willing - “No”
