# Supplementary figures and images for: Development of a novel clinical support tool for active surveillance of low risk papillary thyroid cancer
Source: Front Endocrinol (Lausanne). 2023 Sep 11;14:1160249. doi: 10.3389/fendo.2023.1160249 (PMC10520546; doi:10.3389/fendo.2023.1160249)

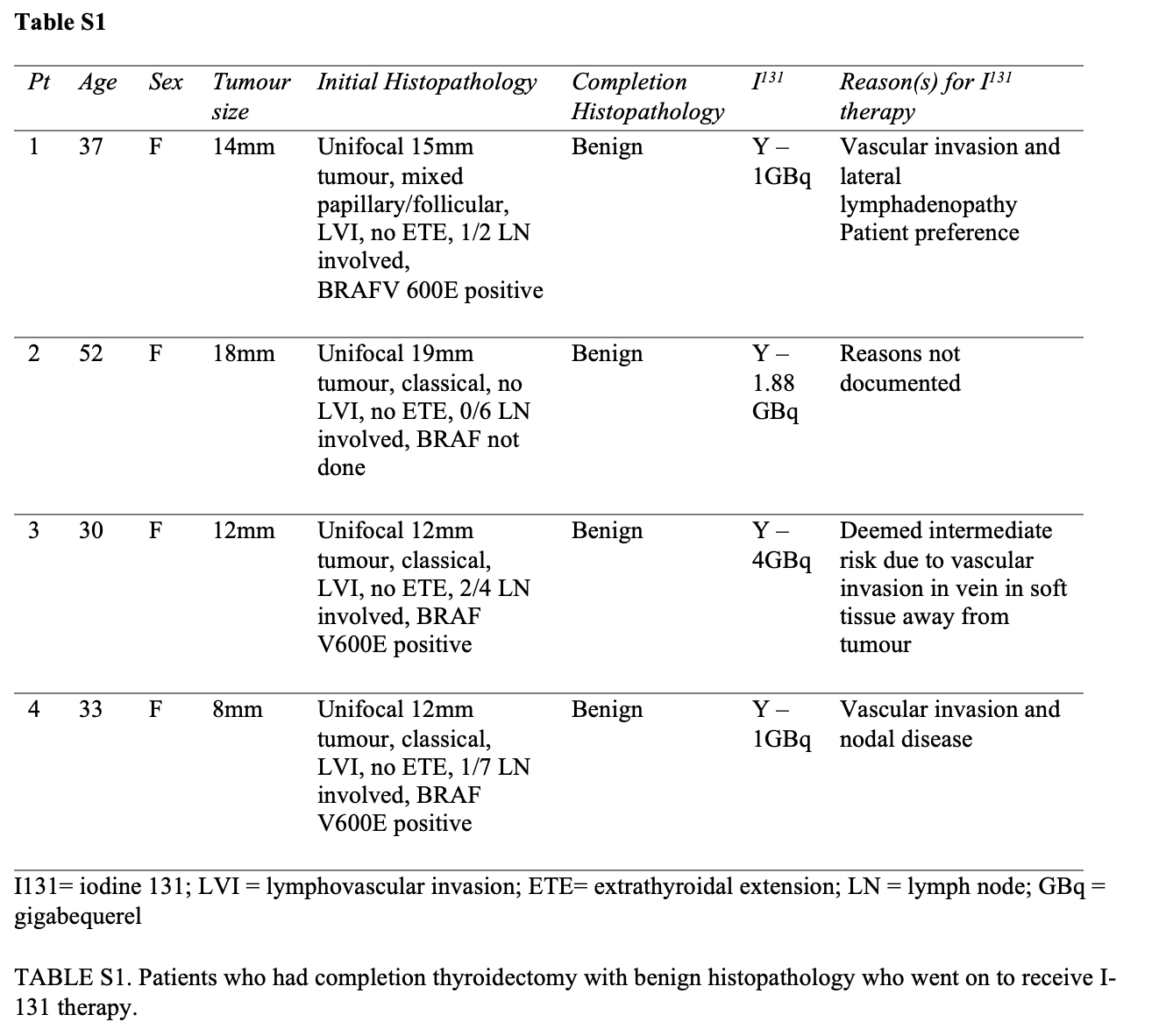

Supplement: Supplementary file 2 [file Table_1.docx]
